# Water makes glass elastically stiffer under high-pressure

**DOI:** 10.1038/s41598-018-30432-7

**Published:** 2018-08-08

**Authors:** Motohiko Murakami

**Affiliations:** 1 Department of Earth Sciences, ETH Zürich, Zürich, 8025 Switzerland; 2 Department of Earth and Planetary Materials Science, Graduate School of Science, Tohoku University, Sendai, 980-8578 Japan

## Abstract

Because of its potentially broad industrial applications, a new synthesis of elastically stiffer and stronger glass has been a long standing interest in material science. Various chemical composition and synthesis condition have so far been extensively tested to meet this requirement. Since hydration of matter, in general, significantly reduces its stiffness, it has long been believed that an anhydrous condition has to be strictly complied in synthesis processes. Here we report elastic wave velocities of hydrous SiO_2_ glass determined *in-situ* up to ultrahigh-pressures of ~180 gigapascals, revealing that the elastic wave velocities of hydrous glass unexpectedly show the rapid increase with pressure and eventually become greater than those of anhydrous glass above ~15 gigapascals. Furthermore, anomalous change in the velocity gradient at ~100 gigapascals, probably caused by the change in Si-O coordination number from 6 to 6+, was also found at ~40 gigapascals lower pressure condition than that previously reported in anhydrous silica glass, implying that water is a highly effective impurity to make SiO_2_ glass much denser. This experimental discovery strongly indicates that hydration combined with pressurization is highly effective to synthesize elastically stiffer glass materials, which offers a new insight into the fabrication of industrially useful novel materials.

## Introduction

Glasses with extraordinary optical transmittance and isotropy, high chemical-, corrosion-, and heat-resistance, and low thermal expansion have been widely used in various industrial fields such as chemical, electronic, automobile, architectural and medical industries. Although there has recently been a very strong demand for downsizing, lightening, and thinning of such glasses for further advanced applications especially to the microelectronics, the fatal flaw intrinsic to the glass that is too brittle for such machining bottlenecks to further potential growth of those industrial applications. Improving the strength and toughness of the glass has thus long been awaited for future manufacture of industrially useful splinterless glass products.

Glass materials are generally considered to be elastically deformed under certain stress conditions until the fracture stress is reached, which results in the crack initiation, and then rapid fracture occurs along with the propagation of such cracks. Therefore, in order to study toward making the splinterless glasses, it is essentially important to understand the sequence of fracture mechanisms of the glass from the elastic deformation to the rapid fracture through initiation and propagation of cracks. It has been well known that the actual fracture strength for glass materials normally exhibits almost two orders of magnitude smaller than the theoretical fracture strength estimated from the interatomic bonds of the glass. The most widely accepted reason for that is that the potential presence of microcracks on the glass surface induce the significant stress concentration equivalent to the theoretical fracture stress at the tiny crack tip even if the apparent/applied stress is very small^1^.

A number of studies on the strength of the glasses have thus far been focused on the elimination or suppression of the initiation and propagation of the microcracks at the glass surface, and thermally or chemically strengthening methods, which create the glass surface to be in a state of compression tension, have successfully increased the practical fracture strength of the glass by a factor of ~3–8^2–5^.

Since the hydration of glasses, in general, result in less stiffer materials^6–9^, it has long been believed that an anhydrous condition has to be strictly complied to prevent the softening of the glass products in the course of synthesis processes. Although improving the elastic stiffness intrinsic to the glasses is obviously one of the most critical issues to evaluate the fracture characteristics which dominantly controls the very initial stage of the fracture behavior, systematic understanding of the effect of chemical composition on the elasticity of the glasses has been still limited.

Unlike the crystals, glasses without having a periodic arrangement of atoms in structure can possess arbitrary chemical compositions, which enable to provide the new improved properties by freely changing the variety or amount of chemical components. On the other hand, such infinite divergence in composition of the glasses might have conversely been inhibiting the systematic studies so far. Among such wide varieties of glasses in composition, a SiO_2_ glass is thought to be one of the most fundamental and important network-forming simple oxide glasses^10,11^ with features for broader practical applications, which would thus be a very suitable analogue material for exploring the elastic properties of the glass.

Despite a few unusual cases, pressurizing the glass materials essentially enhances its elastic stiffness, and thus pressurizing has been thought to be promising way to make elastically strong glasses. However, due to the technical hurdles, the experimental studies were only limited to very low pressure conditions^12,13^, which prevents from precise understanding of the effect of pressures.

In addition, it has been few studies to explore the combined effects of chemical composition and pressure on the elasticity of the glass. Since the elastic wave velocity data of a material under pressure directly reflects its elastic moduli and density, one of the most straightforward ways to evaluate the elasticity change of the glass material with pressure would be to determine its elastic wave velocities under pressures. We have recently proven that the Brillouin scattering spectroscopy combined with diamond anvil cell (DAC) high-pressure apparatus is highly feasible for the determination of elastic wave velocities of the glass materials under extreme pressure condition exceeding to 200 GPa^14–16^.

To clarify this issue, ultrahigh-pressure elasticity measurements of hydrous silica glass were performed up to ~180 GPa using newly-developed high-pressure Brilluoin scattering interferometry combined with DAC.

## Results

**Figure 1:**
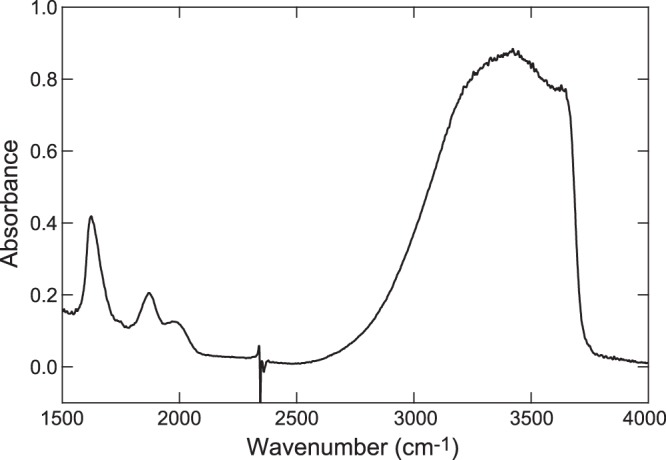
Infrared absorption spectrum of hydrous SiO_2_ glass. The water content of the hydrous SiO_2_ glass was determined based on the formalisation and parameterisation by Davis *et al*.^17^. Hydroxyl concentrations in terms of ppm OH by weight was calculated by the modified equation of the Beer-Lambert law as follows, *C*_ppm OH_ = (*A*/*L*)(1/ε_OH_)(1/1000)*17*(10^6^)/ρ, where *A* is the maximum height of an optical absorbance band at 3673 cm^−1^; ε_OH_ is the molar absorptivity for that band; ρ is the density of the silica glass (=2.2 (g/cm^3^); and *L* is the thickness of the sample. We adopted the representative value of 77.5 (*l*mol^−1^cm^−1^) as the ε_OH_^17^. *A* was found to be 0.63 after the linear baseline correction of the spectrum.

Hydrous SiO_2_ glass was prepared by mixing of SiO_2_ glass (*Suprasil*-*P*) and H_2_O liquid water. The mixture was placed in a piston-cylinder apparatus under water saturated condition at 0.8 GPa and 1200 °C for 24 hours and subsequently quenched. The water content of the synthesized glass was determined by the Fourier transform infrared microscopic (*FTIR*) measurements. The IR spectra were obtained from doubly polished glass sample with 40 μm thickness. The IR light emitted by a light source through a Ge-coated KBr beam splitter is focused on a sample by means of Cassegrain mirrors. This light passes through a sample, to a MCT (HgCdTe) detector. Several hundreds scans were accumulated with 4 cm^−1^ resolution to obtain IR absorption spectra of the sample. We estimated the water content of the glass sample with the use of IR absorption spectrum (Fig. 1) and on the basis of the Beer-Lambert law^17^. The result shows that the silica glass contains ~1.6 wt% hydroxyl (OH).

**Figure 2:**
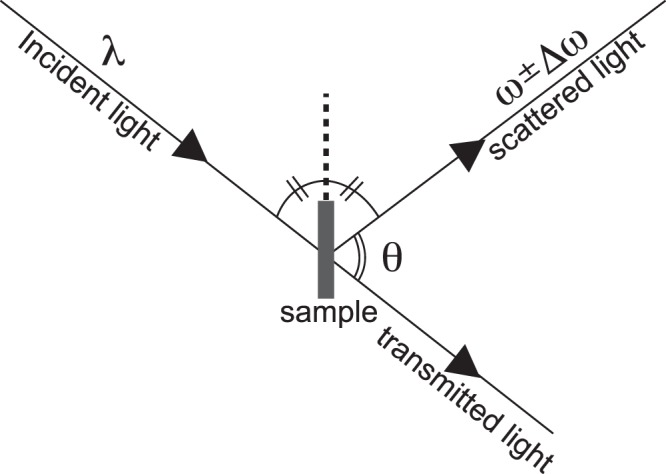
Schematic drawing of the experimental configuration for the Brillouin scattering spectroscopy in a symmetric scattering geometry. λ, incident beam wavelength; θ, the external scattered angle; ω, frequency of Rayleigh scattered component; Δω, frequency of inelastically scattered component (Brillouin frequency shift).

Elastic wave velocities of hydrous SiO_2_ glass were measured under ultrahigh-pressures by means of Brillouin scattering spectroscopic system with DAC at room temperature at Tohoku University^16^. A diode-pumped laser (λ = 532 nm) was used as a probe beam, and scattered light was analysed by the tandem-type multi-pass Fabry-Perot interferometer. Spot size of the focused laser onto the sample in a DAC was ~10 μm. Brillouin scattering data were obtained in the 50° symmetric platelet scattering geometry (Fig. 2). Hydrous SiO_2_ glass powder was pre-pressed to make thin pellet with a thickness of ~40 μm, and the sample pellet was placed into a sample chamber with a diameter of ~50 μm fabricated in the pre-indented rhenium gasket. For generating high-pressures, we used beveled diamond anvils with 150 μm culet size. No pressure-transmitting medium was used. Ruby fluorescence pressure scale^18^ and Raman *T*_2g_ mode of the diamond anvil^19^ were applied to determine the pressure, and pressure distribution within the laser focused area were consistent within 0.6 GPa in average. We collected thirty-eight Brillouin spectra of the compressed hydrous SiO_2_ glass from 12 to 177 GPa with the measurement time ranging from 40 minutes to 96 hours typically depending on the pressure condition. Elastic velocity at each pressure is then calculated with knowledge of the Brillouin frequency shift, laser wavelength and external scattering angle.

**Figure 3:**
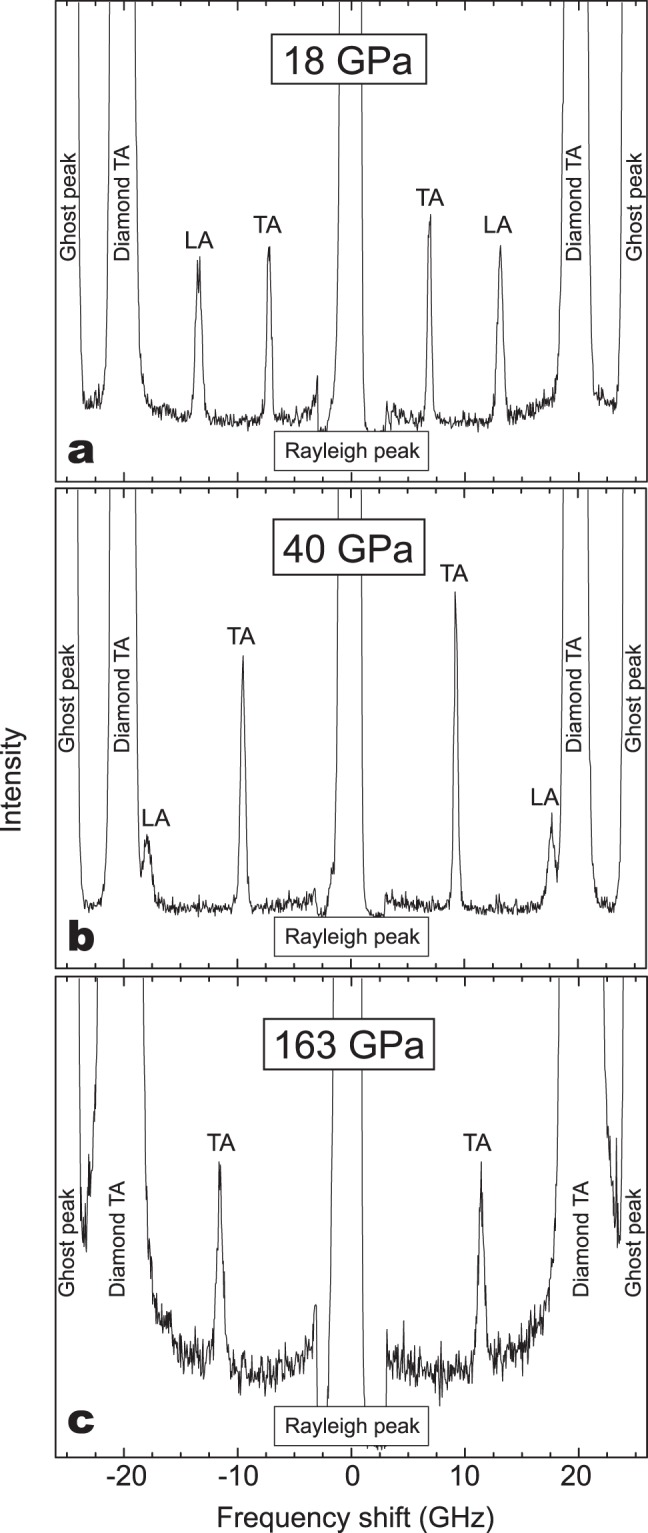
Brillouin scattering spectra of hydrous SiO_2_ glass under high-pressures. The Brillouin scattering peaks from the sample can be found to shift to higher absolute value of the frequency shift with increasing pressure of 18 GPa (**a**), 40 GPa (**b**) and 163 GPa (**c**). *TA* and *LA* indicate transverse and longitudinal acoustic modes of the Brillouin scattering shift, respectively. Ghost peaks are artefacts of the interferometry method used.

Figure 3 shows the Brillouin spectra of the hydrous SiO_2_ glass under high-pressures. As shown in Fig. 3b, the Brillouin peaks from longitudinal acoustic modes of the hydrous SiO_2_ glass become overlapped with those of transverse acoustic modes of the diamond anvils above ~40 GPa, which prohibits the determination of longitudinal acoustic waves velocities above ~40 GPa (Fig. 3b). The sharpness of the Brillouin peak has been believed to be very sensitive to the hydrostaticity in the sample. Given that significant pressure-induced peak broadening was not observed (Supplemental Table 1), it is highly indicative that the remarkable change in hydrostaticity did not occur with pressure.

**Figure 4:**
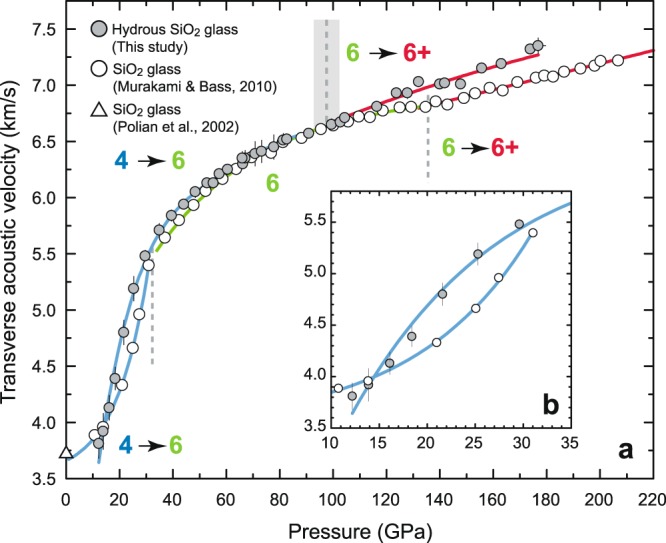
Transverse acoustic wave velocities of hydrous SiO_2_ glass under high-pressures. The open symbols show the previous experimental data on anhydrous SiO_2_ glass at high-pressure up to 207 GPa by Murakami & Bass (2010)^14^ and at ambient pressure by Polian *et al*.^33^. (**a**) shows the whole pressure range we explored, and (**b**) shows the enlarged plots from 10 to 35 GPa. Polynomial curve fitting (from fourth to ninth) was applied to all data to estimate quantitative trend change in the velocity profile with pressure, showing the significantly good fit (average *R*^2^_*adj*_ = 0.9969). To determine the pressure condition that the trend change in the velocity occurs, we follow the same method as described in our previous literature^15^, and this point was found to be at 98 (±4) GPa. The most probable coordination number of Si is shown in colored number within each pressure regime divided by the vertical dot lines which shows the approximate pressure boundary.

Transverse acoustic wave velocity profile with pressure up to 177 GPa for hydrous SiO_2_ glass is shown in Fig. 4 together with that of anhydrous SiO_2_ glass up to 207 GPa that we have recently reported^14^. We found that, at lower pressure condition below ~30 GPa, the elastic wave velocity profile of the hydrous SiO_2_ glass that initially show the lower velocity at ~12 GPa exhibits very steep trend, and the velocities becomes significantly greater than those of anhydrous SiO_2_ glass by up to ~10% velocity contrast (Fig. 4b). Above ~30 GPa, the velocity contrast subsequently becomes smaller with pressure approaching to ~60 GPa, and the velocities of hydrous SiO_2_ glass shows almost the same velocities as those of anhydrous SiO_2_ glass within the experimental uncertainties up to ~100 GPa. However, surprisingly, the velocity profile of hydrous SiO_2_ glass shows anomalously steeper trend above ~100 GPa, and the velocities again becomes greater with pressure than those of anhydrous SiO_2_ glass by up to ~4.3% at 177 GPa.

The overall trend change in velocity with pressure in the present study is quite consistent with that of previous experimental works on the other silicate glasses such as MgSiO_3_^15^ or SiO_2_-Al_2_O_3_ glasses^16^ determined under very high pressures up to ~200 GPa. Such trend change can be basically explained by the sluggish structural transition from 4- to 6-fold coordinated silicon atoms at lower pressure and above 6-fold coordination under pressure condition above ~100 GPa. Recent theoretical and experimental studies^20,21^ also support this interpretation. We also found that the pressure condition at which the anomalous velocity increase occurs above ~100 GPa (hereafter referred to as “the inflection pressure”) is ~40 GPa lower than that of SiO_2_ glass. Given that the addition of 20 mol% Al_2_O_3_^16^ or 50 mol% MgO^15^ into SiO_2_ glass only reduce the inflection pressure by 24 GPa and 7 GPa respectively, the fact that the addition of ~1.6 wt% of OH into SiO_2_ glass reduces the inflection pressure by ~40 GPa strongly indicates that H_2_O is a highly effective impurity to make the SiO_2_ glass much denser under high pressure.

It is well known that adding a few % of water into the glasses leads to a significant elastic softening of the glasses, which has been considered to be critical deterioration in glass quality in terms of stiffness. For example, previous experimental results have shown that the addition of ~3 wt% of water into the silicate glasses decreases their elastic moduli by up to ~6%^22^. This is also the case with the silicate minerals^23–25^, and a ~16% decrease in elastic moduli has previously been reported in the silicate mineral with ~2 wt% of water^26^. Regarding the glass, one of the primary factors that led to this significant decrease in elasticity by water is that H_2_O acts as the network modifier like CaO or Na_2_O, which cuts the Si-O bonds of Si-O network structure and form the non-bridging Si-OH groups in SiO_2_ glass.

Contrary to such conventional view, the present study reveals that small impurity of water by ~1 wt% into SiO_2_ glass in combination with the application of pressure at least above ~15 GPa leads to significantly greater elastic wave velocity than that of the anhydrous SiO_2_ glass. Given the fact that a few % of water does not significantly change the densities of various silicate glasses^22^ and the effect of water in reducing the melt/glass density is known to be markedly diminished under pressure^22,27^, this unusual elastic behavior strongly implies the water makes SiO_2_ glass more elastically stiffer under pressure. This uncommon characteristic under pressure is most likely due to the fact that the pressure would change the role of H_2_O from network modifier to network former, which is extremely rare example because it has been thought that even adding the considerable amount of MgO or Al_2_O_3_, having both features of network modifier and network former at least under ambient condition, into SiO_2_ glass by up to 50% would not make the elastic wave velocities exceed to those of SiO_2_ glass under high pressures at least above ~25 GPa^15,16^. H_2_O is thus an extremely unique component that works as the network former under high pressure and makes the silicate glasses elastically stiffer.

It has been widely accepted that the phase transformation kinetics in crystals is drastically affected by the pressure, temperature and the presence of dissolved water^28^. Although the kinetics of the structural transformation of glass materials is not yet fully understood especially under high-pressure condition, a few pioneering experimental works indicate that the high-temperature actually enhances the structural densification of SiO_2_ glass under relatively low pressure condition below 10 GPa^11^. Given those facts, the pressure-induced anomalous structural evolution of the hydrous SiO_2_ glass observed in the present study might be partly caused by the possible effect of dissolved water on kinetics in structural change.

SiO_2_ glass has been known as one of the most hardest oxide glasses which is able to synthesize inexpensively in terms of material cost without any special procedures or additives, and therefore it is widely used for the varieties of industrial products. The present study found that, under pressurizing condition, the elastically much stiffer silicate glasses than SiO_2_ glass can be synthesized at equivalent pressure by adding an ordinary substance of H_2_O, which has been thought as the typical impurity to make the materials softer. From the point of view of practical use, it is essentially important to consider whether this stiffening feature under pressure can be related to the permanent densification, or not. The mechanism of the permanent compaction of the glass materials, however, still remains unknown especially under ultrahigh-pressure condition. According to the previous relatively low-pressure experimental works, it has been believed that the permanent compaction of the SiO_2_ glass takes place around 9–13 GPa most likely due to the structural transformation in the intermediate-range order relating to the network structure consisting of SiO_4_ tetrahedra^29^. Sato & Funamori, 2008 further explored the higher-pressure structures of silica glass approaching to ~100 GPa based on the *in-situ* synchrotron X-ray diffraction measurements^30^. Although it remains speculative, they claimed that the SiO_2_ glass should behave elastically from 13–20 GPa with stable state of 4-fold coordinated Si, and that, between 20–40 GPa, the irreversible structural change would occur in the short-range order associated with the transformation from tetrahedrally coordinated Si to octahedral coordination of Si. If this interpretation were the case, it would be possible to think that the permanent densification occurs in the hydrous SiO_2_ glass as well under high-pressure regimes owing to the structural transformations from 4- to 6-fold, and from 6-fold to 6+ coordination of Si. Ultimately, this issue should be confirmed by the thorough decompression experiment of the glass recovered from extreme pressure condition. Although there might be still a technical hurdle for mass production owing to relatively high pressure condition, several experimental attempts to secure the large volume of the sample under high pressure have been successfully performed^31^. This experimental discovery would thus open a new research/industrial avenue for synthesis procedure of the elastically hard glass materials.

## Methods

In the Brillouin scattering spectroscopy measured under the symmetric platelet scattering geometry, the elastic wave velocity, *V* can be determined using following relation:

$$V={\rm{\Delta }}\omega \lambda /2sin(\theta /2)$$

where Δω is the Brillouin frequency shift, λ is the wavelength of the incident beam, and θ is the external scattered angle. To determine the precise external scattered angle, we measured a borosilicate crown optical glass (BK7) as a standard calibrant under ambient condition, whose elastic velocities were previously reported. All measurements were performed in a symmetric platelet scattering geometry. More experimental details can be found elsewhere^15,32^.

## Electronic supplementary material

Supplementary Table 1
