# Supplementary material for: Water makes glass elastically stiffer under high-pressure
Source: Sci Rep. 2018 Aug 8;8:11890. doi: 10.1038/s41598-018-30432-7 (PMC6082893; doi:10.1038/s41598-018-30432-7)
Supplement: Supplementary file 1 — Supplementary Table 1 [file 41598_2018_30432_MOESM1_ESM.pdf]

Water makes glass elastically stiffer under high-pressure

Motohiko Murakami

Supplementary Table 1.

Experimentally determined pressure (*P) and* shear velocity (V_S) of hydrous silica glass, and half width at half maximum (*HWHM*) of Brillouin peaks from TA mode of the sample.

P (GPa)	*P error* (GPa)	*Vs* (km/s)	*Vs error* (km/s)	*HWHM* (GHz)	*HWHM error* (GHz)
12.2	0.1	3.81	0.13	0.68	0.01
13.9	0.0	3.92	0.16	0.19	0.01
16.1	0.0	4.13	0.11	0.26	0.01
18.4	0.1	4.39	0.10	0.19	0.01
21.6	0.1	4.80	0.11	0.21	0.01
25.3	0.1	5.19	0.11	0.18	0.01
29.6	0.2	5.48	0.05	0.16	0.01
34.9	0.2	5.71	0.06	0.19	0.01
39.5	0.0	5.84	0.05	0.16	0.01
44.1	0.0	5.94	0.03	0.25	0.03
48.4	0.2	6.05	0.05	0.17	0.00
52.8	0.1	6.13	0.04	0.17	0.01
55.1	0.4	6.13	0.03	0.17	0.01
57.4	0.2	6.21	0.03	0.18	0.00
60.5	0.3	6.25	0.02	0.17	0.01
66.0	1.0	6.35	0.06	0.25	0.01
66.2	0.0	6.30	0.04	0.20	0.01
67.0	0.3	6.35	0.07	0.20	0.01
70.8	0.3	6.39	0.11	0.21	0.01
73.2	0.1	6.41	0.10	0.19	0.01
77.8	0.0	6.45	0.11	0.19	0.01
81.3	0.0	6.51	0.06	0.25	0.02
82.7	0.0	6.52	0.01	0.50	0.04
90.9	0.5	6.57	0.02	0.44	0.03
99.4	0.1	6.65	0.05	0.37	0.03
102.7	0.2	6.67	0.02	0.51	0.04
104.4	2.7	6.71	0.03	0.53	0.03
116.4	0.3	6.81	0.01	0.37	0.03
123.7	0.2	6.93	0.02	0.32	0.03
127.8	0.4	6.93	0.05	0.36	0.05
132.0	0.3	7.03	0.01	0.34	0.04
140.0	0.4	7.01	0.04	0.26	0.03
141.8	1.3	7.02	0.02	0.39	0.11
147.8	0.2	7.01	0.03	0.39	0.05
155.7	0.3	7.15	0.00	0.42	0.04
163.0	0.1	7.19	0.00	0.30	0.02
173.9	0.7	7.32	0.04	0.42	0.04
176.8	2.8	7.35	0.07	0.28	0.04
